# Physical mapping in highly heterozygous genomes: a physical contig map of the Pinot Noir grapevine cultivar

**DOI:** 10.1186/1471-2164-11-204

**Published:** 2010-03-26

**Authors:** Simone Scalabrin, Michela Troggio, Marco Moroldo, Massimo Pindo, Nicoletta Felice, Giuseppina Coppola, Giacomo Prete, Giulia Malacarne, Raffaella Marconi, Giorgia Faes, Irena Jurman, Stella Grando, Taco Jesse, Cinzia Segala, Giorgio Valle, Alberto Policriti, Paolo Fontana, Michele Morgante, Riccardo Velasco

**Affiliations:** 1 Dipartimento di Matematica e Informatica, Università di Udine, Via delle Scienze 206, 33100 Udine, Italy; 2 Istituto di Genomica Applicata, Parco Scientifico e Tecnologico di Udine "Luigi Danieli", Via J Linussio 51, 33100 Udine, Italy; 3 IASMA Research and Innovation Centre, Foundation Edmund Mach, Via E Mach 1, 38010 San Michele all'Adige (TN), Italy; 4 Dipartimento di Scienze Agrarie ed Ambientali, Università degli Studi di Udine, Via delle Scienze 208, 33100 Udine, Italy; 5 Keygene NV, PO Box 216, 6700 Wageningen, The Netherlands; 6 Dipartimento di Biologia, Università degli Studi di Padova, Via Ugo Bassi 58b, 35121 Padova, Italy

## Abstract

**Background:**

Most of the grapevine (*Vitis vinifera* L.) cultivars grown today are those selected centuries ago, even though grapevine is one of the most important fruit crops in the world. Grapevine has therefore not benefited from the advances in modern plant breeding nor more recently from those in molecular genetics and genomics: genes controlling important agronomic traits are practically unknown. A physical map is essential to positionally clone such genes and instrumental in a genome sequencing project.

**Results:**

We report on the first whole genome physical map of grapevine built using high information content fingerprinting of 49,104 BAC clones from the cultivar Pinot Noir. Pinot Noir, as most grape varieties, is highly heterozygous at the sequence level. This resulted in the two allelic haplotypes sometimes assembling into separate contigs that had to be accommodated in the map framework or in local expansions of contig maps. We performed computer simulations to assess the effects of increasing levels of sequence heterozygosity on BAC fingerprint assembly and showed that the experimental assembly results are in full agreement with the theoretical expectations, given the heterozygosity levels reported for grape. The map is anchored to a dense linkage map consisting of 994 markers. 436 contigs are anchored to the genetic map, covering 342 of the 475 Mb that make up the grape haploid genome.

**Conclusions:**

We have developed a resource that makes it possible to access the grapevine genome, opening the way to a new era both in grape genetics and breeding and in wine making. The effects of heterozygosity on the assembly have been analyzed and characterized by using several complementary approaches which could be easily transferred to the study of other genomes which present the same features.

## Background

With a production of 67.1 millions tons obtained from 7.9 millions hectares in 2004, grapevine (*Vitis vinifera *L.) is by far the most important fruit tree crop in the world http://www.oiv.org. In comparison to other fruit crops, it shows a particularly wide range of uses: fresh, dried, transformed in juice and obviously wine. At present, its importance goes beyond the economic level, being wine associated in many cases with national cultures and life style. Moreover, red wines are thought to provide health benefits thanks to secondary metabolites such as resveratrol, a strong antioxidant supposed to provide health benefits [1].

Despite the obvious economic relevance, most of the grape cultivars grown today were selected several centuries ago from spontaneous crosses and then vegetatively propagated. Still today, the release of new varieties is a considerably slow process. This is mainly due to some peculiar genetic and physiological features which indeed are shared with most of the tree crops and which significantly hamper the adoption of modern breeding strategies. The main difficulty for genetic analysis is due to the fact that the genome of grape is highly heterozygous, even if the species is actually mainly autogamous [2,3]. Furthermore, grape is a perennial plant with a relatively long life cycle, which makes breeding expensive, due to space constraints, and time consuming [4].

Because of the lack of breeding activity, the improvements in grape production have relied mainly on agronomy, including a strong use of chemicals that may have a negative impact on human health and environmental quality. One of the main goals in grapevine breeding is therefore to obtain varieties naturally resistant to various pathogens [4].

As a tool for modern breeding, genome-wide physical maps are assuming a more and more central role because they are useful for many purposes. First of all, even if the technologies for genome sequencing have undergone a remarkable progress during the last years, genome assembly largely depends on physical mapping. The clone-by-clone approach always requires physical mapping, because a minimal tiling path of clones must be selected to separately sequence them [5]. Nevertheless, also the whole-genome shotgun (WGS) strategy greatly benefits from physical data and indeed it is still unclear if it could alone be sufficient to produce a linearly ordered set of sequences [5]. A second application of physical maps is the large-scale isolation of genes using the positional cloning approach. In fact, this is the only strategy which can be used to identify genes which can be genetically mapped, but whose function is unknown - which is true for most traits of agronomic importance [6]. Eventually, physical maps can also be used for several other kinds of studies *e.g.*, to compare genomes and to understand their size and complexity [5,7,8].

Taking into account all these aspects and considering that grape is an 'orphan species' from a genetic perspective, it is clear that an integrated physical map would be of great utility both for research *stricto sensu *and for practical applications, such marker-assisted breeding.

Grapevine is also attractive for future sequencing efforts, being a diploid organism which can be easily crossed and selfed, and having a relatively small genome of only about 475 Mb [9]. Furthermore, grape has several unique features including a novel shoot architecture and non-climateric fruits producing a number of secondary metabolites such as colour pigments, tannins and flavour compounds. Finally, as a basal family of the Eurosids, the *Vitaceae *are also interesting for comparative studies [10]. Thus, grape can be regarded as a model organism.

Whole-genome physical maps have been constructed for several organisms such as *Caenorhabditis elegans *[11], *Drosophila melanogaster *[12], human [13], mouse [14], and chicken [15]. In plants, physical maps are available for *Arabidopsis thaliana *[16,17], sorghum [18], rice [19], soybean [20], black cottonwood [21], apple [22], *Prunus *[23], and the grapevine cultivar Cabernet Sauvignon [24]. Most of these physical maps have been obtained by adhering to different approaches, mainly by marker hybridization [25] and BAC fingerprinting [26].

Sequence Tagged Site content-based methods are laborious and require an extremely high density of markers. This piece of information is rather difficult to obtain for the large of majority of plant genomes, and especially for orphan species [5]. On the contrary, fingerprinting-based strategies are better suited to genomes which are not well explored e.g., grape. Since these techniques are also more amenable to high-throughput processing, they have been chosen in the last years for the majority of physical mapping projects.

Due to their relevance, the techniques of fingerprinting have undergone a fast evolution during the last two decades. Indeed, a range of methods has been proposed which vary in several parameters, but especially in the detection of the fingerprinting fragments. These techniques can be divided into three groups, which are the agarose-gel based, the acrylamide-gel based and the capillary sequencer based methods. The last set of approaches, also known as High Information Content Fingerprinting (HICF), shows a substantial increase both in the throughput and in the sensitivity of the process [27,28].

Recently, it has been debated on which HICF methodology would work best. Xu *et al. *[28] evaluated five different fingerprinting techniques and concluded that a two-enzymes approach was more effective than the others. This result is rather counterintuitive, since three-, four- or five-enzymes based approaches provide more information and should be able to better discriminate between false positive and false negative errors. Indeed, according to Nelson and coworkers [29], the most effective protocol is the one developed by Luo *et al. *[30], which indeed is based on the use of five restriction enzymes. This method has been used in this work after adapting it to grape.

Recently, important progress has been made in genomics research of various fruit trees and woody plants. The draft genome sequence of the black cottonwood tree, *Populus trichocarpa*, has been completed using a whole genome shotgun approach [31], and a physical map is available as well [21]. Genome-wide physical maps of the apple and peach genomes have been recently released [22,23]. To end up with, the whole genome sequences of two different genotypes of grapevine are now available, one of which based on the inbred line PN40024 [32], and the other one based on the heterozygous genotype Pinot Noir [33]. Both of these sequencing efforts have been carried out following a WGS approach, therefore without the need of producing an earlier physical map.

**Figure 1:**
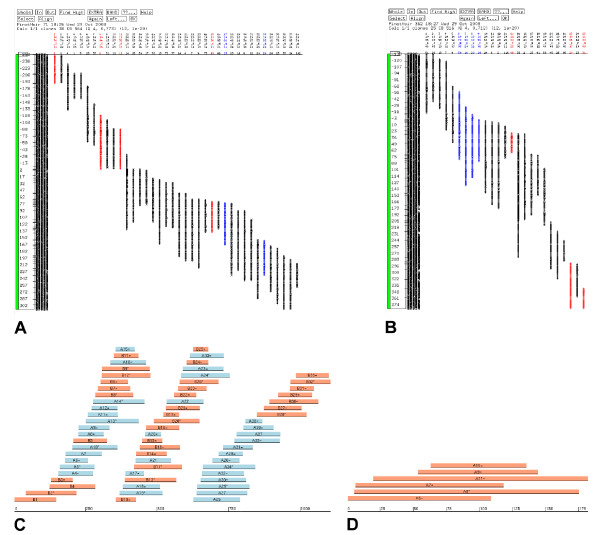
**Effect of heterozygosity on the assembly of the physical map**. **a| and b| **CB Maps of two real contigs of the grape physical map ('a' corresponds to contig number 69 and 'b' to contig number 354) showing the so called 'scissoring effect', one of the main consequences of heterozygosity. On both contigs a polymorphic SNP marker has been physically mapped by BAC-pooling first, followed by PCR determination of the BAC clones containing each of the two alleles. The clones on which one allele maps have been highlighted in red and the ones on which the other allele maps in blue. It is interesting to observe that the two alleles of the same SNP marker map onto different clones which are not overlapping each other. The problem which affects the assembly of these two contigs has been called 'scissoring effect'. Basically, the clones within a contig tend to split apart, with the ones belonging to one allele at one extremity and the ones belonging to the other allele at the other extremity. As a consequence, the affected contigs show an expansion in the CB map size. **c| and d| **Two plots depicting simulated contigs from the *in silico *simulation at 42% sequence divergence, where the clones belonging to one allele have been highlighted in orange and the ones belonging to the other allele in blue. In 'c' the 'scissoring effect' is particularly evident on the right extremity, while the other part of the contig shows a more complex situation. Indeed, the scissoring effect can be locally more or less important and this is thought to be related to the variation in the levels of heterozygosity along the contig/chromosome. This situation is particularly consistent with the pictures 'a' and 'b'. The contig displayed in 'd' shows instead a different situation, being made up by clones which belong only to one allele (in orange). This scenario is consistent with the cases of allele testing in which the two alleles hybridize onto separate contigs (see text).

Here we report a BAC-based physical map of the grape genome and its integration with the genetic map, a molecular tool which will change the approach to the genetics and breeding of this crop. As already stated, grapevine cultivars are highly heterozygous [3,24]. Physical maps constructed till now present almost no (*e.g.*, mouse and *Arabidopsis*) or very low heterozygosity (*e.g.*, human). The only exceptions are black cottonwood, *Prunus*, apple, and the Cabernet Sauvignon grapevine cultivar. Black cottonwood and apple maps are based on agarose fingerprints and the effects of heterozygosity with such method are smoothened by the higher tolerance allowed in the assembly. Beside that, a specific study about the effects of heterozygosity on the map assembly has been performed only in the case of black cottonwood [21]. On the other hand, the map of *Prunus *has been obtained with the same fingerprinting method chosen for grape, but other differences make the comparison quite difficult. In fact, the mapping effort in peach has not been completed yet and the clones fingerprinted so far (4.3× genome equivalents), are biased towards the expressed regions of the genome. Moreover, two BAC libraries were used in this case, one obtained from a diploid genotype and the other from a haploid one, which could have reduced the impact of heterozygosity. Finally, concerning heterozygosity, the map of Cabernet Sauvignon, obtained with the same protocol as the Pinot Noir map, lead to similar results: incorrect order of the BAC clones within a contig, producing apparent duplications of *loci *in the physical map and assembly of BAC clones corresponding to two different haplotypes into separate contigs. See Figure 1 and the discussion below for further details, including an *in silico *simulation related to the effects of heterozygosity on a map assembly.

Assessing the effects of heterozygosity on physical maps is important, because there is reason to believe that it could affect their correct assembly, similarly to what has been observed for the DNA sequence assembly in *Ciona savignyi *[34]. Therefore we propose our work as a useful model for any future physical mapping effort on heterozygous genomes, which are very common among plants.

## Results and discussion

A total of 49,104 BAC clones from two BAC libraries representing approximately 11.4 genome equivalents [35] (Table 1) of the red wine cultivar Pinot Noir, one of the most widely grown in the world, were fingerprinted, adapting to grape a fluorescence-based, high-throughput method developed for wheat and rice [30]. The 38,983 clones that remained after processing the raw fingerprint data for background removal and identification of failed, empty, and contaminated clones (see methods) were assembled using the software FPC 8.9 [36] and following an iterative method that was first used in maize [37] to minimize the effects of contamination and repetitive bands and to improve the overall quality of the map. The final assembly resulted in 1,804 contigs estimated to span about 888 Mb (3,372 BACs remained unassembled *i.e.*, singletons; Table 2), including 2,310 BACs (6%) of difficult resolution after fingerprinting (Questionable or simply Q clones), distributed in 662 Q contigs. The total length of the contigs corresponds roughly to 1.87 times the estimated 475 Mb size of the grape genome [9]. This indicates that the genome size may have been underestimated [38], or that contig overlaps have gone undetected (false negative overlaps), but may also result from high DNA sequence heterozygosity *i.e.*, from the presence of two very different haplotypes in a single diploid genome. As already stated, physical maps have been constructed so far for species with no (*e.g.*, inbred species such as mouse, rat, rice, Arabidopsis, and maize) or very low heterozygosity (*e.g.*, human), with the exception of black cottonwood, apple, and *Prunus*. Grapevine cultivars are highly heterozygous [3]; Adam-Blondon (personal communication), by comparing 180 kb of sequenced genomic DNA (cv. Cabernet Sauvignon) from both homologous chromosomes, observed 8.9% of variable nucleotides of which a large proportion (88%) was due to indels that were observed at an average frequency of one every 554 bases. Furthermore, the recent sequencing of the genome of grape cv Pinot Noir showed an average 11.2% of allelic difference between homologous chromosomes by considering both single nucleotide polymorphisms (SNPs) and indels, with important variations according to the genomic region [33]. DNA polymorphisms between homologous regions introduce variations in the fingerprints of allelic BAC clones, that could result in two different contigs being assembled for a given chromosomal region. In the above mentioned regions of the grapevine cv. Cabernet Sauvignon genome the sequence divergence between the two homologous chromosomes resulted on average in 37% non shared fingerprint bands as assessed by *in silico *digestion, with marked differences between regions (Adam-Blondon, personal communication). As the software FPC is not designed to recognize and bring together "allelic" contigs, an expansion of the physical map due to sequence heterozygosity is a realistic possibility. The expansion of the whole genome DNA sequence assembly as a result of high levels of polymorphism has been observed in *Ciona savignyi *[34]. *V. vinifera *is the first highly heterozygous organism for which a BAC contig HICF physical map has been constructed. A previous work in maize, where extremely high levels of sequence polymorphism due to transposable element insertions and nucleotide substitutions exist within the species [7], to construct contig maps of specific regions showed that haplotypic differences between chromosomes in a hybrid could often result in separate contigs being assembled for each chromosome [7]. Nevertheless, the other physical maps constructed on heterozygous genomes show important differences with respect to the case of grapevine and therefore they can only partially be taken as a comparison. To begin with, in the cases of apple and black cottonwood an agarose-based approach was chosen. According to Nelson and Soderlund [27], repetitive bands in agarose fingerprinting should be less of a problem than in sequencer-based methods because of the bigger size of restriction fragments and to the fact that small indels are not detected. The same pattern could be observed also in the case of heterozygosity. The fact that in the maps of black cottonwood and apple an expansion of about 1.2 fold the genome was observed compared to 1.87 fold in the Pinot Noir could confirms this hypothesis. However, in black cottonwood it was often observed that two different physical contigs aligned to the same region of a sequence contig: this fact suggests the existence of haplotype-specific contigs in the BAC map [21]. Furthermore, if only a single haplotype was considered for each of the co-aligning contigs anchored to the sequence, then the overall genome size estimate represented by the map showed almost no expansion. To sum up, the expansion of the black cottonwood map is, at least partially, due to the formation of allelic physical contigs, but if compared to grapevine the rate of expansion is reduced. The reason for this difference in the expansion could be found in the fingerprinting technique, but also in the different parameters used for the map assembly.

**Table 1:** Sources of BACs fingerprinted to develop the *Pinot Noir *physical map

Libraries	Cloning site	Mean insert size	No. of clones fingerprinted	Genome coverage
grp01 (Evry)	*Hind*III	93 kb	23,040	4.6 ×

grp02 (Keygene)	*Hind*III	125 kb	26,064	6.8 ×

Total (Both libraries)	*Hind*III	110 kb	49,104	11.4×

**Table 2:** Status of the grape cv. *Pinot Noir *physical map assembly.

Number of clones fingerprinted	49,104
Number of clones used for map assembly	38,983

Number of singletons	3,372

Number of contigs	1,804

>200 clones	1

101-200 clones	14

51-100 clones	139

26-50 clones	350

10-25 clones	503

3-9 clones	557

2 clones	240

Unique bands in the contigs	807,776

Total physical length of the contigs (Mb)*	888

Number of Q clones	2,310

Number of Q contigs	662

*Each fingerprinted band was estimated to represent an average of 1.1 kb. The estimate was based on the average insert size of the BAC clones divided by the average number of bands per clone used for contig assembling

In this work, the impact of DNA sequence heterozygosity on the physical map assembly was evaluated by using different approaches. First, 35 SNP markers were selected and BAC clones containing the alternative alleles were identified to ascribe them to the two haplotypes. All of the SNP markers had been genetically mapped to a unique position within the genome, to avoid the potentially confounding effect of genome duplications. However, it is to be considered that genome duplications in grapevine should not pose a big problem for the assembly of a physical map. In fact, even if grapevine appears to be an ancient hexaploid [32], the last round of duplication within its genome took place before the separation from black cottonwood, that is about 60-65 Myrs ago [31,32]. Therefore, the impact of duplicated genomic regions (or homeology) on the map is likely to be very reduced in grapevine, and not as important as it is for instance in soybean [38].

The analysis carried out on this set of SNPs showed that both haplotypes mapped to the same contig in 13 (37%) cases ('A' class), while each haplotype mapped to a different contig in 10 (29%) cases ('B' class). In the remaining 12 (34%) cases ('C' class), BACs ascribed to the same haplotype were found on more than one contig and showed several different patterns. In 6 out of those 12 cases, the two haplotypes were found on the same contig, but one or both of the two alleles did also map to other contigs or singletons. In the other 6 cases, the two haplotypes were always found on separated contigs, but one or both of the two alleles were found on more than one contig or singleton. Altogether, these 35 polymorphic markers were found on 59 physical contigs, which corresponded to 1.7 contigs per marker. Moreover, if the same calculation was performed on the 'C' class', the value rose to 2.3 contigs per markers. The finding that allelic BAC clones *i.e.*, deriving from each of the two haplotypes, mapped frequently to different contigs is consistent with the increased size of the physical map when compared to the estimated genome size and shows that DNA sequence heterozygosity results in significant differences in BAC fingerprints. The FPC software resolves such difficulties in a conservative mode increasing contig number. Furthermore, this evidence is perfectly in agreement with the results obtained in the physical map of black cottonwood [21]. Even when the BAC clones from the two haplotypes assembled in a single contig (Figure 1a, b) their relative positioning within the contig was often not accurate, with the clones from each haplotype locally assembling separately rather than overlapping each other. This local assembly problem, which we named "scissoring effect", was not observed in the black cottonwood assembly.

Second, assemblies were performed using *in silico *constructed BAC fingerprints that simulated differences due to increasing levels of DNA heterozygosity between the two allelic haplotypes (Table 3). The number of Q clones increased in parallel to the increase in the DNA differences between the haplotypes. This supports the conclusion that the high frequency of contigs with Q clones shown in Table 2 derives largely from the high level of DNA heterozygosity. When the differences in fingerprint bands between the two haplotypes reached 42% we also observed a sharp increase in the total map size and in the number of contigs assembled. Multiple contigs were often assembled, that frequently contained BAC clones from a single haplotype (Figure 1d). When the BAC clones from the two haplotypes assembled in a single contig (Figure 1c) we often observed the same local assembly problem ("scissoring effect") that we observed in the real contigs that causes an artifactual map expansion. These two phenomena together accounted for the significant expansion of the total map size of the region that reached up to a 40% increase, showing a similar trend to the one that we observe in the real assembly of the grape map. Still, it is to be considered that the real fingerprints represent a much more complex situation, due to the perturbing effect of several factors like the presence of residual contaminations, unremoved background peaks, chimeric clones, and the complexity of the whole genome. These aspects are likely to be responsible for the difference between the 1.87× expansion found in the real assembly and the 1.4× expansion obtained from the simulated data. In any case, *in silico *simulations agree with the data from our grape map assembly and with the analysis of the BAC clones containing the alternative alleles reported above in terms of map expansion and the separation of haplotypes into different contigs. A difference of 42% in the fingerprint bands obtained from the two haploptypes is realistic given that it requires 4.2% average nucleotide sequence divergence between them.

**Table 3:** Computer simulations of the effects of haplotype polymorphism on contig assembly using FPC.

% different fingerprint bands	% average sequence divergence	Consensus band units for entire region	Map coverage of the region	Number of contigs	% Questionable clones
0	0	1,172.5 ± 39.9	1.0 ± 0.0	1.1 ± 0.3	0.0 ± 0.0

25	2.5	1,252.4 ± 89.0	1.1 ± 0.1	1.2 ± 0.4	6.0 ± 4.9

42*	4.2	1,681.6 ± 129.4	1.4 ± 0.1	2.2 ± 1.0	18.8 ± 8.5

50	5.0	1,717.7 ± 206.5	1.4 ± 0.2	2.2 ± 0.9	24.7 ± 9.2

For each level of sequence divergence average and standard deviation values were computed for each of the parameters out of ten replicates. Coverage values are the ratio between the CB map length of the simulated contig and the CB map length of the original region (1,192 CB units).

* The average of 42% difference in fingerprint bands results from two areas without differences (containing 100 bands each, simulating small regions of identity by descent) and 50% difference in the rest of the region. In the other two cases differences were randomly distributed across the region.

To integrate the physical map with the genetic map, two complementary strategies were adopted. The first was based on developing genetic markers from BAC-end sequences and placing them on the linkage map. 316 markers were produced using this approach. 48 of them were uninformative because derived from clones that were not assembled into contigs and the remaining 268 assigned contigs to specific positions on the linkage map. The second strategy made use of BAC pools [39] constructed according to Klein *et al. *[18]. A six dimensional pooling geometry represented the best compromise to achieve a high efficiency in unambiguous marker assignment to BAC clones, while reducing considerably the number of PCR assays. A total of 24,576 BAC clones were pooled in six distinct directions (or six unique coordinate axes) to generate 184 pools. Primers for 133 microsatellite markers and 174 EST markers were used to screen the BAC pools. 15 unique AFLP primer combinations that identified 149 markers on the Pinot Noir genetic map [40] were also used. 25 SSR and 36 EST markers could not be assigned unambiguously to BAC clones (See methods) and were discarded from further analyses. An average of 4.9 BAC clones were identified for ESTs, 3.6 for SSRs, and 2.0 for AFLP markers (Table 4). AFLP markers identified a single BAC more frequently than SSR or EST markers: this can be explained by the high stringency adopted during AFLP screenings of pools, which led to discard faint bands that could have resulted in marker mis-assignment to BACs.

**Table 4:** Results of BAC pools screening with molecular markers.

	ESTs	SSRs	AFLPs
Markers not assigned to BAC clones*	36	25	13

*False positive or not fingerprinted BAC clones*	16	7	8

*Not amplified in one of the six pools or over-represented in BAC clones*	20	18	5

Markers assigned to BAC clones:	138 (4.9 clones*)	108 (3.6 clones*)	149 (2.0 clones*)

*Class I: identified two or more BACs assembled in a single contig*	82	54	39

*Class II: identified only one BAC in a single contig*	36	38	69

*Class III: identified singletons (unassembled BACs)*	6	7	31

*Class IV: identified multiple BACs assembled into distinct contigs*	14	9	10

Tested	174	133	162

Following BAC screening, each single putatively positive BAC clone has been picked and tested to confirm the primary pool screening result.

* average number of BAC clones identified for each marker type

580 markers in total were used for the integration of genetic and physical maps, including 89 SSRs, 116 ESTs, 107 AFLPs, and 268 BAC-end markers. A total of 436 contigs were assigned a position on the genetic map, covering 341.6 Mb. As expected, the average size of unassigned contigs was considerably smaller than the one of positioned contigs (Table 5). 104 contigs, covering 102.0 Mb, were anchored by two or more markers, whereas 332, covering 239.6 Mb, were anchored by one marker. For only 2 contigs, covering 1.6 Mb, more than one location was determined on the genetic map because two of the markers located on them mapped at distinct locations on the genetic map. This conflict might depend from errors present either in the genetic or in the physical map, but more likely from genome duplications. Although grapevine chromosomes pair as bivalents, and the species has disomic inheritance [41], the genome of grapevine should be considered polyploid (paleopolyploid [42,43]) because organized in more than 11 chromosomes. This is not surprising because angiosperms have a propensity to polyploidization [44], to the point that recent reviews speak of all angiosperms [45], or 80% of them [46], being to some extent polyploid. Data for sequenced dicot genomes indicate that both Arabidopsis and poplar may have undergone 3 rounds of duplications [31,44,47], with the most recent one in poplar leading to high sequence similarity between duplicated regions. Thus, some DNA markers may identify multiple loci on the physical map, only one of which may have been genetically mapped, leading to contigs being assigned to multiple genetic locations. Obviously, one of such two locations should correctly correspond to a given contig.

**Table 5:** Statistics of the grape cv. *Pinot Noir *physical map by linkage group

Linkage group	No. of contigs	Coverage (Mb)	Average contig size (Kb)
1	22	16.442	747.4

2	23	18.086	786.3

3	18	16.723	929.1

4	20	16.204	810.2

5	21	19.363	922.0

6	24	19.514	813.1

7	29	23.517	810.9

8	27	19.104	707.6

9	16	9.922	620.1

10	26	17.971	691.2

11	20	16.114	805.7

12	21	18.910	900.5

13	28	22.111	789.7

14	24	21.627	901.1

15	17	12.814	753.8

16	20	10.416	520.8

17	18	17.927	995.9

18	37	28.286	764.5

19	25	16.570	662.8

Unassigned	2	1.600	800.0

Total contigs assigned to markers	436	341.621	783.5

Unassigned contigs	1,368	534.832	391.0

Total contigs	1,804	888.553	492.5

**Figure 2:**
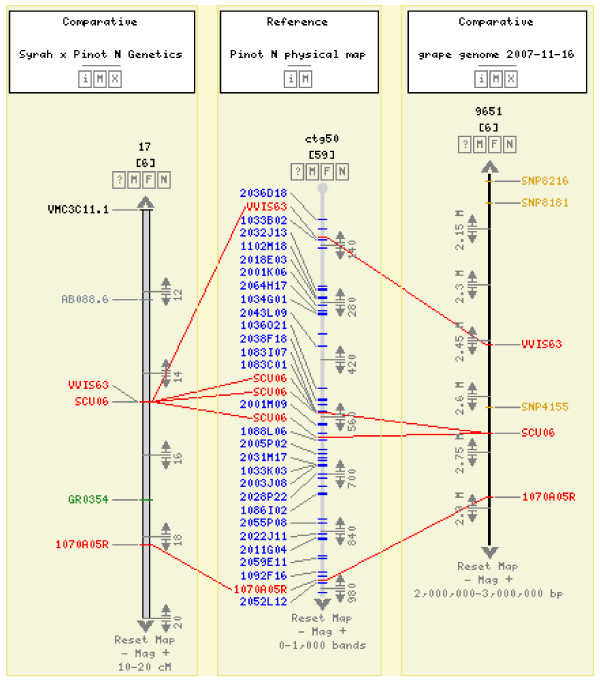
**Comparison of the physical contig 50 (CB units 0-1,000) with linkage group 17 (10-20 cM interval in the genetic map) and the cluster 9651 (2-3 Mb) from the genome sequence assembly of Pinot noir (CMap viewer available at http://genomics.research.iasma.it)**. Correspondences among marker *loci *in the genetic map, BAC clones in the physical contig, and marker positions in the sequence are displayed with solid lines.

The physical map assembly was validated at several scales. Molecular markers were used to assess the overall quality of our map following an *a posteriori *approach as described by Meyers *et al. *[5]. When two or more genetic markers identified BAC clones that were included in a single contig, we observed a very good colinearity of markers in the physical map and in the dense genetic map derived from the cross Syrah X Pinot Noir (see above). If all the contigs anchored to the genetic map by two or more markers were considered, it was possible to observe that in 104 out of 108 (96%) cases the markers were very close on the genetic map. Subsequently, a similar survey was undergone by taking into account only the molecular markers developed from BAC end sequences. In fact, these markers offer the best opportunity to validate the physical map assembly since their location on the physical map is unambiguously determined. The results obtained in this second case are very similar to the previous ones. In fact, in 26 out of 27 such cases (96%) the genetic markers developed from the same contig did co-map or map very closely on the genetic map. Then, the assembled sequences of the Pinot Noir cultivar [33] offered the possibility of controlling BAC order in the physical map on a smaller scale. An example is provided in Figure 2 where three genetic markers present in a region of the linkage map of chromosome 6 are positioned in the same order in the DNA sequence cluster 22067 and in the physical contig 189. In addition, it is worth to observe a methodological detail: BACs containing the same marker were correctly assigned to the contig. However, they are not always overlapping as expected, most probably because of the "scissoring effect" that we previously ascribed to the heterozygosity of Pinot Noir which contributes to the physical map expansion (see Web FPC at http://genomics.research.iasma.it).

Afterwards, the sequence of the genome of the inbred line of grape PN40024 [32] was also used for an assessment of the whole physical map. 30,158 BAC-ends obtained from the 'Pinot Noir' were positioned on the PN40024 chromosomes through a stringent blast (e-value 1E-50, with at most three hits allowed to discard repeats): 11 contigs out of 1804 (0.6%) including at least two groups of at least three BAC-ends each aligning to different linkage groups were considered chimeric. In most of the cases such contigs were linked by a single chimeric clone (Table 6).

**Table 6:** Chimeric clones detected by BES alignment versus the genomic sequence (linkage groups between parentheses) and by counting putative intra-plate contaminations (high number of clones from a single plate, in parentheses, in the same contig)

Contig	Putative chimeric clone(s)	Detection method	E-value (when merged)
14	1047_O11, 1040_J15	BES alignment (7, 14)	

31	1101_K03, 2051_H20	BES alignment (2, 7)	

61	1037_H06	BES alignment (4, 5)	

329	2024_E09, 1093_O01	BES alignment (2, 18)	3e-25

458	1087_L01	BES alignment (1, 14)	

552	2010_D07	BES alignment (2,18)	

961	1038_L10	BES alignment (6, 16)	4e-37

994	1086_L13	BES alignment (4, 14)	3e-26

1424	1084_I11, 1099_A14	BES alignment (4, 16)	2e-25

2207	All clones from plate 1044	Intra-plate (1044)	

2811	1039_G08, 1041_B15	BES alignment (18, 19), Intra-plate (1039)	

3130	Uncertain	BES alignment (7, 10, 14)	

## Conclusions

We have developed a resource that facilitates the access to the grapevine genome in several ways. By one hand, this tool can be used to easily create a link between genetic maps and genomic sequences, thus facilitating tasks such as positional cloning or QTL mapping. By another point of view it could also become useful for comparative purposes when studying other species belonging to the family of Vitaceae.

The second relevant aspect which has been studied in this work is the effect of heterozygosity on the construction of fingerprinting-based physical maps. Experimental and *in silico *approaches have been undertaken and have given comparable results, thus representing an example of how the use of simulated data can help the study of genomes. Furthermore, the description of the effects of heterozygosity in physical mapping could be particularly useful for further studies of genomes showing the same features.

It looks like the assembly could be improved by the utilization of new map assembly algorithms that explicitly deal with the presence of two haplotypes regardless of the levels and patterns of heterozygosity.

## Methods

### BAC library construction and BAC pooling

*Hind*III partially digested HMW DNA from *V. vinifera *cv. Pinot Noir clone 115 was used to construct two libraries. The first was obtained as described by Adam Blondon *et al. *[35] and consisted of 23,424 clones (4.6× genome coverage) stored into 384-well microplates. The average insert size and the percentages of empty clones and plastidial DNA were controlled as well [35]. The second library was produced at Keygene (Wageningen, The Netherlands). DNA was ligated into the pIndigoBAC vector and ligation mixes with less than 4% of empty clones, less than 4% of cpDNA contamination and insert size >125 kb were selected. After transformation into *E. coli *strain DH10β, 26.112 colonies were picked up (6.8× genome coverage). In total, 49,104 clones were available for fingerprinting. Average insert sizes for the two libraries are described in Table 1.

BAC pooling followed the strategy proposed by Klein *et al. *[18] 24,576 BAC clones (5× genome coverage) were arranged in a stack which was sampled in six distinct ways and a total of 184 pools were obtained.

### BAC fingerprinting and BAC-end sequencing

BAC DNA was isolated and fingerprinted adapting to grape the technology already developed for wheat and rice [30]. BAC clones maintained in 384-well microplates were preinoculated in 384-well microplates containing 70 μl 2× LB medium plus chloramphenicol (12.5 mg/ml) per well, inoculated in 96-well plates containing 1.2 ml of the same medium per well, and grown at 37°C with shaking at 250 rpm for 10-15 h. BAC DNA was isolated using the 96-well Unifilter plates (Whatman 7700-0062) following the procedure described by Luo and coworkers [30] and dissolved in 20 μl of ddH_2_O.

A total of 5 μl of a solution containing 5 units each of *Bam*HI, *Eco*RI, *Nde*I, *Xba*I, and *Hae*III restriction enzymes; 1× NEBuffer 2 (New England Biolabs, Boston, MA); 2 μg BSA; 2 μg DNase-free RNase; and 0.1% β-mercaptoethanol was added to 15 μL of the BAC DNA solution and the digestion was carried out at 37°C for 3 h in a 384-well PCR plate. Subsequently, 3 μL of labelling solution made up of 1.5 μL SNapShot Multiplex Ready Reaction Mix (Applied Biosystems, Foster City, CA), 0.8 μL Tris-HCl 100 mM, pH = 9.0, and 0.7 μL ddH_2_O were added to each well and the plates were incubated at 65°C for 1 h.

The BAC fingerprinting reaction was precipitated in ethanol, resuspended in 10 μL of ddH_2_O and filtered through Sephadex using the 384-well genCLEAN plates (Genetix, New Milton, UK). After cleaning, DNA was resuspended again in 10 μL of ddH_2_O and a mixture of 10.98 μL of Hi-Di formamide and 0.02 μL of Genescan LIZ-500 internal size standard (Applied Biosystems, Foster City, CA) was added to each well. The samples were then denaturated and loaded into an ABI 3730 DNA sequencer (Applied Biosystems, Foster City, CA).

End sequencing of 19,957 BAC clones was performed using 2.5 μL of BAC DNA and 1.0 of Big Dye 3.1 Terminator Ready Reaction Mix (Applied Biosystems, Foster City, CA), for a total reaction volume of 10 μL. Traces were analysed by a pipeline of programs, including Phred, RepeatMasker, Mummer, Cap3, and CrossMatch for quality clipping, clustering, and repeats identification (unpublished data). Some of the unique sequences were then used for BAC anchoring to the linkage groups of the genetic map.

### Data processing and assembly of the physical map

The ABI GeneMapper 3.5 software (Applied Biosystems, Foster City, CA) was first used to analyze all electrochromatograms and to produce a text file (containing areas, heights, and sizes for each peak) for each BAC fingerprint. Then, 809 ribosomal and 941 chloroplastic contaminated clones were removed. Ribosomal and chloroplastic clones were detected through a comparison to previously built-in ribosomal and chloroplastic fingerprint patterns and by blasting the 30,158 BAC-ends versus the ribosomal (e-value 1E-30) and chloroplastic (e-value 1E-200) sequences inferred from the genome assemblies. Then, remaining clones were processed by FPB [48], a PERL script to distinguish between peaks originating from BAC clone restriction fragments and background peaks (*e.g.*, *E. coli *genomic DNA contamination or machine noise) using an iterative procedure[24], to remove empty clones and vector bands and to convert data to FPC format as in Genoprofiler. Finally, 384 and 96 cross-well contaminations were detected with the use of the software GenoProfiler [49]: 1471 adjacent clones sharing at least 50% of their bands were considered cross-well contaminated and removed before the assembly. A further analysis to prove that the *in silico *procedure was sufficient to remove contaminations was performed on contig 71 that is apparently highly contaminated as 19 of its 38 clones originated from only 9 plates. Two analysis were carried out. Initially, BAC-end sequencing and corresponding alignment on the PN40024 sequence[32] revealed that in all cases but 2 there was no contamination since sequences were aligning in a single region of chromosome 1; only two reads had the best hit on a different location (with their mate on the correct place) [see Additional file 1]. Successively, four clones with no sequence or with uncertain BES alignment were picked up from glycerol stock and inoculated in 2× LB Medium with chloramphenicol (12.5 mg/ul) over night, after that each clone was streaked onto 2× LB medium agarose plate with chloramphenicol and grown over night. Six single colonies for each clone were picked and inoculated in 2× LB medium with chloramphenicol over night individually. BAC DNA of all clones were isolated and tested by capillary electrophoresis fingerprinting. Fingerprints produced for each of the six single colonies within each of the individual BAC clone resulted identical.

The contig assembly was carried out based on the software FPC 8.9 and following the approach suggested by Nelson *et al. *[37]. Tolerance was set at 0.4 bp and for the initial build the cutoff value was set at 1E-50. The map was then further assembled in an iterative way, through 6 rounds at successively less stringent cutoffs. Each round consisted in: 1) a 3-step DQing process starting at the same cutoff of the previous assembly and then decreasing the cutoff by 10^5 ^at every successive step and 2) an automatic end-joining step performed increasing the cutoff value of the previous assembly by 10^5 ^times. Finally, contigs were tested with internally developed scripts, to detect intra-plate contaminations: only one contig was seriously affected by such a problem (see Table 6, contig 2207).

### *In silico* assessment of the effects of heterozygosity on BAC contig assembly

We started from the Consensus Band (CB) map of a high quality assembled contig containing 1192 bands. We assumed this to represent the ordered fingerprint bands for the region from a single chromosome/haplotype. We produced the expected CB map of the same region from the homologous/allelic chromosome/haplotype by randomly modifying a certain percentage of bands corresponding to a certain level of sequence divergence. As a first approximation each band produced in our fingerprinting method can be considered to be defined by 10 nucleotides and therefore a randomly distributed sequence divergence of 1% between the two haplotypes is expected to produce a difference in 10% of the fingerprint bands obtained from the two chromosomes/haplotypes. For each of the two chromosomes BACs were created randomly to provide 5× coverage by defining an average insert size (120 bands) and a range of variation (from 50 to 190 bands). Ten replicates of the BACs from the two haplotypes were created for each sequence divergence condition. The assembly was carried out iteratively, as already described for the map, starting from a cutoff value of 1E-50. Averages of the different estimated parameters were then obtained from the 10 replicates.

### Integration between genetic and physical maps

A linkage map of the *V. vinifera *L. cross Syrah x Pinot noir [40] was the basis to anchor genetically fingerprinted BAC clones. In the genetic map, 994 markers were positioned with the help of 94 F_1 _hybrid individuals and grouped in 19 linkage groups covering a total length of 1,245 cM. Linkage group assignment and ordering of loci was established based on TMAP software [50] that finds the maximum likelihood map using an error-compensating model. Details of linkage map construction are provided elsewhere [40].

The integration between physical and genetic maps was carried out in two steps. First, 133 simple sequence repeats (SSRs) and 174 expressed sequence tags (ESTs) mapped markers were used to screen the BAC pools following a hot start amplification strategy [40]. After adding SYBR Gold, samples were run on 1.5% agarose gel. BAC clones hosting SSR and EST markers were identified by a Unix-based application with a web interface. Additional anchor points were provided by screening the BAC pools with the 15 unique amplified fragment length polymorphism combinations [51] used to identify 162 mapped AFLPs. Preamplification and selective amplification were performed as for linkage analysis. AFLP amplification products from BAC pools were analyzed on acrylamide gels along with amplification products from the two parents and the mapping population as a control (AFLP Quant-Pro, Keygene, Wageningen, NL). BACs containing AFLPs were identified as for the other markers. Finally, the genetic position of 316 SNP markers developed from the BAC-ends provided additional anchoring information.

## Abbreviations

AFLP: Amplified Fragment Length Polymorphism; BAC: Bacterial Artificial Chromosome; BES: BACEnd Sequence; EST: Expressed Sequence Tag; HICF: High Information Content Fingerprinting; QTL: Quantitative Trait Locus; SNP: Single Nucleotide Polymorphism; SSR: Simple Sequence Repeat; WGS: Whole Genome Shotgun.

## Authors' contributions

SS wrote the script for fingerprint background removal, removed the contaminations, assembled the map, performed the *in silico *assessment of heterozygosity and the *in silico *validation of the whole map and participated in drafting the manuscript. MT anchored the physical contigs to the genetic map with the help of MP, GC, GM, and SG and participated in drafting the manuscript. MaM constructed the physical map with the help of NF, GP, RM, participated to the map assembly and validation and drafted the manuscript. MP constructed the BAC pools. GF constructed the 'grp01' BAC library. GV performed BES sequencing with the help of IJ. TJ developed AFLP markers with the help of GM and constructed the 'grp02' BAC library. CS performed some bioinformatic analysis and server maintenance with the help of PF. AP contributed to the discussion of the results. MiM and RV conceived the study, participated in its design and coordination and contributed to reviewing the manuscript. All authors read and approved the final manuscript.

## Supplementary Material

Additional file 1 **BAC-end alignment on the PN40024 sequence to disprove contaminations**. contig 71 is apparently highly contaminated as 19 of its 38 clones originated from only 9 plates. To disprove this BAC-end sequencing and corresponding alignment on the PN40024 sequence[32] was carried out. Clones from plate 1070 are contaminated at the library level (they are identical) since corresponding BES align at the same positions. Only two BES hit (with low similarity percentage) on different locations but with their corresponding mate blast hit in the correct location. Click here for file
